# A concept for integrated care pathways for atopic dermatitis—A GA^2^LEN ADCARE initiative

**DOI:** 10.1002/clt2.12299

**Published:** 2023-09-14

**Authors:** Torsten Zuberbier, Amir Abdul Latiff, Xenofon Aggelidis, Matthias Augustin, Radu‐Gheorghe Balan, Christine Bangert, Lisa Beck, Thomas Bieber, Jonathan A. Bernstein, Marta Bertolin Colilla, Alejandro Berardi, Anna Bedbrook, Carsten Bindslev‐Jensen, Jean Bousquet, Marjolein de Bruin‐Weller, Dayanne Bruscky, Betul Buyuktiryaki, Giorgio Walter Canonica, Carla Castro, Natia Chanturidze, Herberto Jose Chong‐Neto, Chia‐Yu Chu, Leena Chularojanamontri, Michael Cork, Roberta F. J. Criado, Laia Curto Barredo, Adnan Custovic, Ulf Darsow, Arben Emurlai, Ana de Pablo, Stefano Del Giacco, Giampiero Girolomoni, Tanja Deleva Jovanova, Mette Deleuran, Nikolaos Douladiris, Bruno Duarte, Ruta Dubakiene, Esben Eller, Batya Engel‐Yeger, Luis Felipe Ensina, Nelson Rosario Filho, Carsten Flohr, Daria Fomina, Wojciech Francuzik, Maria Laura Galimberti, Ana M. Giménez‐Arnau, Kiran Godse, Charlotte Gotthard Mortz, Maia Gotua, Michihiro Hide, Wolfram Hoetzenecker, Nicolas Hunzelmann, Alan Irvine, Carolyn Jack, Ioanna Kanavarou, Norito Katoh, Tamar Kinaciyan, Emek Kocatürk, Kanokvalai Kulthanan, Hilde Lapeere, Susanne Lau, Mariana Machado Forti Nastri, Michael Makris, Eli Mansour, Alexander Marsland, Mara Morelo Rocha Felix, Ana Paula Moschione Castro, Eustachio Nettis, J. F. Nicolas, Audrey Nosbaum, Mikaela Odemyr, Niki Papapostolou, Claudio A. S. Parisi, Sushil Paudel, Jonny Peter, Prakash Pokharel, Luis Puig, Tamara Quint, German Dario Ramon, Frederico Regateiro, Giampaolo Ricci, Cristine Rosario, Cansin Sackesen, Peter Schmid‐Grendelmeier, Esther Serra‐Baldrich, Kristina Siemens, Cathrine Smith, Petra Staubach, Katarina Stevanovic, Özlem Su‐Kücük, Gordon Sussman, Simona Tavecchio, Natasa Teovska Mitrevska, Diamant Thaci, Elias Toubi, Claudia Traidl‐Hoffmann, Regina Treudler, Zahava Vadasz, Ingrid van Hofman, Maria Teresa Ventura, Zhao Wang, Thomas Werfel, Andreas Wollenberg, Ariana Yang, Yik Weng Yew, Zuotao Zhao, Ricardo Zwiener, Margitta Worm

**Affiliations:** ^1^ Institute of Allergology Charité ‐ Universitätsmedizin Berlin Berlin Germany; ^2^ Allergology and Immunology Fraunhofer Institute for Translational Medicine and Pharmacology ITMP Berlin Germany; ^3^ Pantai Hospital Kuala Lumpur Kuala Lumpur Malaysia; ^4^ Allergy Unit 2nd Department of Dermatology and Venereology National and Kapodistrian University of Athens University General Hospital "Attikon" Athens Greece; ^5^ University Medical Center Hamburg‐Eppendorf Hamburg Germany; ^6^ Institute for Health Services Research in Dermatology and Nursing Hamburg Germany; ^7^ Institutul Regional de Gastroenterologie si Hepatologie Cluj‐Napoca Romania; ^8^ Department of Dermatology Medical University of Vienna Vienna Austria; ^9^ University of Rochester Medical Center Rochester New York USA; ^10^ University Hospital of Bonn Bonn Germany; ^11^ Christine Kühne‐Center of Allergy Research and Education Davos Switzerland; ^12^ University of Cincinnati College of Medicine Cincinnati Ohio USA; ^13^ Hospital del Mar Barcelona Spain; ^14^ Instituto de Asma Alergia y Enfermedades Respiratorias Corrientes Argentina; ^15^ MACVIA‐France Fondation Partenariale FMC VIA‐LR Montpellier France; ^16^ Department of Dermatology and Allergy Centre Odense Research Centre for Anaphylaxis (ORCA) Odense University Hospital University of Southern Denmark Odense C Denmark; ^17^ Division of Allergy and Immunology Complexo Hospital de Clinicas Federal University of Paraná Curitiba Brazil; ^18^ Department of Dermatology/Allergology National Expertise Center for Atopic Dermatitis University Medical Center Utrecht Utrecht The Netherlands; ^19^ Federal University of Pernambuco Recife Brazil; ^20^ Division of Pediatric Allergy Koc University Hospital Istanbul Turkey; ^21^ Personalized Medicine, Asthma and Allergy IRCCS Humanitas Research Hospital Rozzano, Milan Italy; ^22^ Department of Dermatology Hospital Universitario Austral Universidad Austral Buenos Aires Argentina; ^23^ Center of Allergy and Immunology Tbilisi Georgia; ^24^ Department of Dermatology National Taiwan University Hospital Taipei Taiwan; ^25^ Department of Dermatology Faculty of Medicine Siriraj Hospital Mahidol University Bangkok Thailand; ^26^ Sheffield Dermatology Research IICD University of Sheffield Sheffield UK; ^27^ Alergoskin Alergia e Dermatologia Santo Andre Brazil; ^28^ Department of Dermatology Centro Universitario FMABC Santo Andre Brazil; ^29^ Hospital del Mar Parc de Salut Mar Barcelona Spain; ^30^ National Heart and Lung Institute Imperial College London UK; ^31^ Department of Dermatology and Allergy Biederstein Technische Universität München Munich Germany; ^32^ Clinical Hospital Tetovo Tetovo North Macedonia; ^33^ Hospital Universitario Austral Pilar Argentina; ^34^ Allergy and Clinical Immunology University of Cagliari Sardinia Italy; ^35^ Section of Dermatology Department of Medicine University of Verona Verona Italy; ^36^ Health Center Skoplje Skoplje North Macedonia; ^37^ Department of Dermatology Aarhus University Hospital Aarhus Denmark; ^38^ Hospital Santo Antonio dos Capuchos Centro Universitário de Lisboa Central Lisbon Portugal; ^39^ Department of Immunology and Allergology Medical Faculty Clinics of Chest Diseases Vilnius University Vilnius Lithuania; ^40^ Occupational Therapy Department Faculty of Social Welfare and Health Sciences University of Haifa Haifa Israel; ^41^ CPAlpha Clinical Research Center Barueri – São Paulo Brazil; ^42^ St John's Institute of Dermatology St Thomas' Hospital London UK; ^43^ Center of Allergy and Immunology Clinical State Hospital 52 Moscow Ministry of Healthcare Moscow Russian Federation; ^44^ Division of Allergy and Immunology Department of Dermatology, Venereology and Allergology Charité – Universitätsmedizin Berlin corporate member of Freie Universität Berlin and Humboldt‐Universität zu Berlin Berlin Germany; ^45^ Hospital Italiano de Buenos Aires Buenos Aires Argentina; ^46^ Dermatology Department Hospital del Mar IMIM Universitat Autonoma y Universitat Pompeu Fabra Barcelona Spain; ^47^ D.Y. Patil University Navi Mumbai Maharashtra India; ^48^ Department of Dermatology Hiroshima University Hospital Hiroshima Japan; ^49^ Department of Dermatology Kepler University Hospital Linz Austria; ^50^ Department of Dermatology University of Cologne Cologne Germany; ^51^ Clinical Medicine Trinity College Dublin Dublin Ireland; ^52^ Research Institute of the McGill University Health Centre Montreal Quebec Canada; ^53^ Department of Dermatology Kyoto Prefectural University of Medicine Kyoto Japan; ^54^ Ghent University Hospital Ghent Belgium; ^55^ Charité‐Universitätsmedizin Berlin Berlin Germany; ^56^ Faculdade de Medicina Hospital das Clínicas Instituto da Criança e do Adolescente Universidade de São Paulo São Paulo Brazil; ^57^ Division of Allergy and Immunology Department of Clinical Medicine University of Campinas Campinas Brazil; ^58^ Salford Royal Foundation Trust ‐ University of Manchester and Spire Manchester Hospital Manchester UK; ^59^ AlergoLife Clinici Rio de Janeiro Brazil; ^60^ Instituto da Criança Rio de Janeiro Brazil; ^61^ Department of Emergency and Organ Transplantation School and Chair of Allergology and Clinical Immunology University of Bari ‐ Aldo Moro Bari Italy; ^62^ Department of Clinical Immunology and Allergy Lyon‐Sud University Hospital CIRI/INSERM U1111 Lyon France; ^63^ Service d'Allergologie et Immunologie Clinique Centre Hospitalier Lyon Sud Hospices Civils de Lyon Pierre‐Bénite France; ^64^ Centre International de Recherche en Infectiologie INSERM U1111 CNRS UMR 5308 UCBL1 ENS de Lyon Lyon France; ^65^ EFA European Federation of Allergy and Airways Diseases Patients' Associations Brussels Belgium; ^66^ President of the Swedish Asthma and Allergy Association Stockholm Sweden; ^67^ Center Civil Service Hospital Kathmandu Nepal; ^68^ Division of Allergy and Clinical Immunology Department of Medicine University of Cape Town Cape Town South Africa; ^69^ Allergy and Immunology Unit University of Cape Town Lung Institute Cape Town South Africa; ^70^ Instituto de Alergia e Inmunologia del Sur Bahia Blanca Buenos Aires Argentina; ^71^ Allergia e Inmunologia Section Hospital Italiano Regional del Sur Bahia Blanca Buenos Aires Argentina; ^72^ Department of Dermatology Hospital de la Santa Creu i Sant Pau Barcelona Spain; ^73^ Centro Hospitalar Universitário de Coimbra Coimbra Portugal; ^74^ Department of Medical and Surgical Sciences DIMEC University of Bologna Bologna Italy; ^75^ Federal University of Parana Curitiba Brazil; ^76^ Department of Dermatology Allergy Unit University Hospital of Zürich Zürich Switzerland; ^77^ Chrstine Kèhne Center for Allergy Research and Education CK_CARE Davos Switzerland; ^78^ King's College London London UK; ^79^ St John's Institute of Dermatology Guys and St Thomas' NHS Trust London UK; ^80^ Department of Dermatology and Allergy Hautklinik und Poliklinik der Universitätsmedizin University Medical Center Mainz Germany; ^81^ Department of Dermatology Faculty of Medicine Bezmialem Vakif University Istanbul Turkey; ^82^ Faculty of Medicine University of Toronto Toronto Ontario Canada; ^83^ Fondazione IRCCS Ca' Granda Ospedale Maggiore Policlinico Milan Italy; ^84^ ReMedika General Hospital Skopje North Macedonia; ^85^ Center for Comprehensive Inflammation Medicine University of Lübeck Lübeck Germany; ^86^ Allergy and Clinical Immunology Holy Family Hospital Nazareth Israel; ^87^ Environmental Medicine Faculty of Medicine University of Augsburg Augsburg Germany; ^88^ Universtiy Leipzig Medical Faculty Leipziger Interdisziplinäres Centrum für Allergologie Leipzig Germany; ^89^ Bnai‐Zion Medical Center Haifa Israel; ^90^ GA^2^LEN Network Berlin Germany; ^91^ University of Bari Aldo Moro Bari Italy; ^92^ Department of Dermatology Second Affiliated Hospital Northwest Hospital Xi'an Jiaotong University Xi'an Shaanxi China; ^93^ Department of Dermatology and Allergy Hannover Medical School Hannover Germany; ^94^ Department of Dermatology and Allergy Ludwig‐Maximilian‐University Munich Germany; ^95^ Department of Dermatology Free University Brussels University Hospital Brussels Brussels Belgium; ^96^ Division of Allergy and Clinical Immunology of University of Sao Paulo São Paulo Brazil; ^97^ Faculty of Medical Sciences of UNICAMP São Paulo Brazil; ^98^ National Skin Centre Singapore Singapore; ^99^ Department of Dermatology First Hospital Peking University Beijing China; ^100^ Medicina Interna y Alergología e Inmunología Clínica Hospital Universitario Austral Pilar Argentina

**Keywords:** atopic dermatitis, eczema, guidance, ICP, integrated care pathways, multidisciplinary, prevention, treatment

## Abstract

**Introduction:**

The integrated care pathways for atopic dermatitis (AD‐ICPs) aim to bridge the gap between existing AD treatment evidence‐based guidelines and expert opinion based on daily practice by offering a structured multidisciplinary plan for patient management of AD. ICPs have the potential to enhance guideline recommendations by combining interventions and aspects from different guidelines, integrating quality assurance, and describing co‐ordination of care. Most importantly, patients can enter the ICPs at any level depending on AD severity, resources available in their country, and economic factors such as differences in insurance reimbursement systems.

**Methods:**

The GA^2^LEN ADCARE network and partners as well as all stakeholders, abbreviated as the AD‐ICPs working group, were involved in the discussion and preparation of the AD ICPs during a series of subgroup workshops and meetings in years 2020 and 2021, after which the document was circulated within all GAL^2^EN ADCARE centres.

**Results:**

The AD‐ICPs outline the diagnostic procedures, possible co‐morbidities, different available treatment options including differential approaches for the pediatric population, and the role of the pharmacists and other stakeholders, as well as remaining unmet needs in the management of AD.

**Conclusion:**

The AD‐ICPs provide a multidisciplinary plan for improved diagnosis, treatment, and patient feedback in AD management, as well as addressing critical unmet needs, including improved access to care, training specialists, implementation of educational programs, assessment on the impact of climate change, and fostering a personalised treatment approach. By focusing on these key areas, the initiative aims to pave the way for a brighter future in the management of AD.

## 1 INTRODUCTION

The Global Allergy and Asthma European Network, GA^2^LEN, originally started in 2004 as the European Union network of excellence in collaboration with EAACI (European Academy of Allergy and Clinical Immunology), is the largest multidisciplinary network of research centres and clinical care in allergy and asthma. The ADCARE group is a sub network within GA^2^LEN for expertise in atopic dermatitis (AD) that collaborates in research and educational activities as well as in exchange of experience in novel and emerging approaches to treat severely affected patients.

AD is a common chronic inflammatory skin disease representing a lifelong disposition with variable clinical manifestations and expression. The prevalence rates might vary between studies depending on the geographical, genetic, and methodological differences. Cross‐sectional surveys report point prevalence, ranging across countries from 2.1% to 4.9% in adults and from 2.7% to 20.1% in children.^1^, ^2^ In The Odense Adolescents Cohort Study, AD persisted into adulthood in 50% of those diagnosed in school age.^3^ A nationwide Norwegian health registry suggests an increase in the incidence rate of paediatric AD, especially among children younger than 1 year. During the study period, more than 1 in 6 children younger than 6 years had, at some point, been affected by AD.^4^ This represents a significant burden of the disease, warranting effective, efficient, and broadly applicable management strategies that appreciate the complex and variable nature of AD.^5^

AD is a systemic inflammatory condition, including filaggrin deficiency‐induced skin‐barrier disruption and microbiome alteration. Targeting the pathogenesis of AD, a multifaceted approach, including skin hydration, measures to strengthen skin barrier integrity, topical anti‐inflammatory and antipruritic therapy, antibacterial measures, and the elimination of exacerbating factors, can help achieve disease control and prevention of comorbidities. However, many factors limit consistent adherence to treatment plans, such as concerns about side effects, difficulty following time‐intensive and complex skin care routines, the economic burden of therapies, and challenges with lifestyle modifications. Patients with AD have sleep problems very commonly and are also at higher risk of mental health disorders, such as attention‐deficit/hyperactivity disorder, anxiety, depression, disorder of behaviour, autism, and suicide. The visible and chronic nature of AD impacts the quality of life and can contribute to psychological stress, which in turn is a known trigger of itch and skin flares, creating a challenging vicious cycle. Furthermore, bullying is an undesirable consequence of AD that impairs the quality of life.^6^, ^7^, ^8^

**FIGURE 1:**
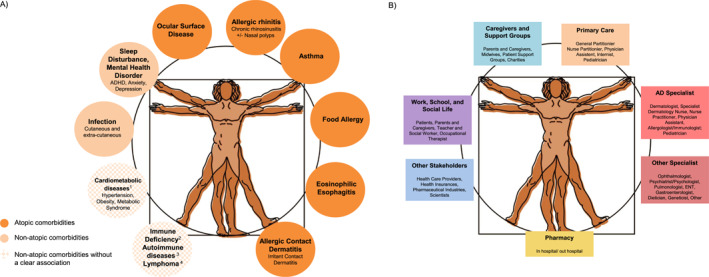
(A) More than skin diseases—comorbidities in AD.^9^, ^10^, ^11^, ^12^, ^13^ ^1^At present, US and Asian data indicate cardiometabolic diseases as a comorbidity of AD, while data from European patients do not support this. Lymphoma is controversially discussed—it is possible that the ‘association’ to AD is based on a misdiagnosis of early stages of CTCL.^14^ ^2^For example, hyper IgE syndromes, WAS and WAS‐like conditions; IPEX and IPEX‐like conditions, CBM‐opathies (CARD11 deficiency, CARD14 deficiency, MALT1 deficiency). ^3^New data indicate autoimmune comorbidities in adults with AD, for example, rheumatoid arthritis, inflammatory bowel disease, and alopecia areata.^15^, ^16^ ^4^Lymphoma is controversially discussed—it is possible that the ‘association’ to AD is based on a misdiagnosis of early stages of CTCL. (B) The AD multidisciplinary team. Please note the country‐specific differences, for example, in some countries, children are usually not seen by the GP during their first years of life but by primary care pediatricians. Hence, pediatricians as well as AD specialists are included as primary care providers. AD, atopic dermatitis; CTCL, cutaneous T cell lymphoma.

A multidisciplinary concept of management and care is needed, considering atopic and non‐atopic comorbidities, aiming for the detection and identification of atopic status, the elimination of exacerbating factors, a fast and effective management of exacerbations as well as long‐term disease control (Figure 1A,B).^17^, ^18^, ^19^, ^20^, ^21^ Accordingly, approaches have been developed in appreciation of the complex interplay among biological, psychological, behavioural, and dietary factors and the wide range of knowledge, skills, and support that patients and families require to effectively manage and cope with this condition.^6^ In consideration of the complex pathophysiology and heterogenous clinical phenotype of AD, more individualised preventative and therapeutic strategies are desirable.^22^

A comprehensive consensus‐based S2k‐guideline for the treatment of children and adults with AD was published as a joint interdisciplinary European project, including physicians from all relevant disciplines as well as patients.^23^ This guideline was upgraded to the S3 level and published in 2022.^24^, ^25^

While evidence‐based guidelines form the basis of AD management, treatment strategies that are used in daily practice are far from guidelines and show significant variation in different jurisdictions or geographical regions. Integrated care pathways (ICP) not only consider different guidelines but can also fill the gaps by providing an expert discussion result based on clinical practice experience of real‐life patient treatment journeys. ICPs offer structured multidisciplinary plans for patient management and have the potential to enhance guideline recommendations by combining interventions and aspects from different guidelines, integrating quality assurance and describing co‐ordination of care.^26^ Hence, ICPs can also consider the different contexts of lower‐ and middle‐income countries as reflected in national guidance.^27^

AIRWAYS ICPs are an example of a multidisciplinary approach to reduce the burden of chronic respiratory diseases, their mortality and multimorbidity, and in the long term to promote active and healthy aging (AHA).^28^, ^29^ The non‐governmental organisation Allergic Rhinitis and Its Impact on Asthma (ARIA) has promoted the integration of its recommendations in ICPs using mobile technology to reinforce self‐management and the implementation of guidelines.^30^, ^31^, ^32^ Following this successful example, a similar strategy of digitally enforced ICPs has been proposed for the setting of AD, to translate guidelines into clinical practice and to treat AD in the context of allergic comorbidities including asthma and food allergy as well as non‐allergic comorbidities such as inflammatory bowel disease and psychological disorders through the coordination between multidisciplinary teams. This publication represents the result of the GA^2^LEN ADCARE initiative based on three conference meetings.

## 2 OBJECTIVES

The general objective of the AD‐ICP working group is to provide a pragmatic and practical support to optimally manage the disease and its comorbidities globally.

AD‐ICPs do not duplicate existing professional guidelines or national prevention programs but aim to strengthen them where appropriate and to help improve adherence to guideline recommendations by translating them into practice, integrating aspects from different guidelines and adjusting them to real‐world conditions in a dynamic way. ICPs also contain information on how to combine therapies for AD and related diseases.

A holistic approach is strived to improve multidisciplinary communication, including primary care, to improve clinician‐patient communication and patient satisfaction, to empower patients and their caregivers, and to engage them following the concept of shared decision making. Accordingly, AD‐ICPs are designed to be carried out by a multidisciplinary team including the support of technology‐assisted patient activation by mobile health tools to enhance self‐management and adherence to guidelines and to serve as a platform for patients to share their experiences.

The detailed objectives, challenges, and unmet needs in the management of AD have been published separately.^33^

## 3 DEVELOPMENT OF THE AD ICPs

### 3.1 Expert discussion

GA^2^LEN ADCARE has taken the lead, informing upfront EAACI, EADV and WAO about the initiative and asking for their involvement in the future, requesting them to send delegates. Other societies will be informed and asked for involvement at a later stage.

The core of the AD‐ICP working group consists of speakers at an online conference held on 26 March 2020. Based on the results of the discussion of several specific working subgroups, the ICPs were then comprised following a structure with boxes indicating the different levels at which certain knowledge and interventions are required.

A second meeting was held on the 12 and 13 of August 2021 with dedicated workshops regarding different topics. Afterwards the document was circulated with all GAL^2^EN ADCARE centres.

### 3.2 Stakeholders

The document involves all stake holders, including the patients, pharmacists, nurses, general practitioners and pediatricians, specialist, tertiary referral centres, the hospitals, academic research institutions, the pharmaceutical industry, and patient organisations. Additional stakeholders not involved in the preparation of the document but included as discussion partners are healthcare institutions, healthcare providers and policy makers.

The document also supports the EU efforts on healthy and active aging (AHA) with GA^2^LEN being a partner in the EIP initiative on AHA.

## 4 ATOPIC DERMATITIS INTEGRATED CARE PATHWAYS

Due to variable clinical manifestations, the variety of available treatments, and in order to become comfortable to take over responsibility for the treatment of their chronic condition, patients and their caregivers need clear and easy‐to‐understand strategies for their individual needs that will allow them to assess, ask, adjust and act.^6^

Different levels of support are available to assist patients in the management of their disease. Figures 2A–H and 3 outline the ICPs for AD involving all stakeholders and self‐management aspects for supporting patients with AD. Patients can enter the ICPs at any level depending on AD severity, resources available in their country, and economic factors such as differences in insurance reimbursement systems. While the AD‐ICPs aim to include as many stakeholders as possible, country‐specific differences need to be considered, such as the role of nurse practitioners, which does not exist in all countries. Also, in some countries, children are usually not seen by the GP during their first years of life but by primary care pediatricians.^41^ Also, a large proportion of all patients with AD suffer from a mild form of the disease and could be managed mainly by the GP, primary care pediatrician and nurse practitioners. Pharmacists are a further, valuable source of support, especially for patients with mild disease, and should therefore be more involved in AD care.

FIGURE 2(A–H) ICPs for the management of atopic dermatitis. (A) ICP—overview. (B) Supported self‐management.^20^, ^23^ (C) Pharmacy.^20^, ^23^, ^34^, ^35^ (D) Primary care.^20^, ^23^, ^36^, ^37^ (E) Specialists I.^20^, ^23^ (F) Specialists II.^20^, ^23^ (G) Caregivers and support groups. (H) Work and social life. ICPs, integrated care pathways.

**Figure:**
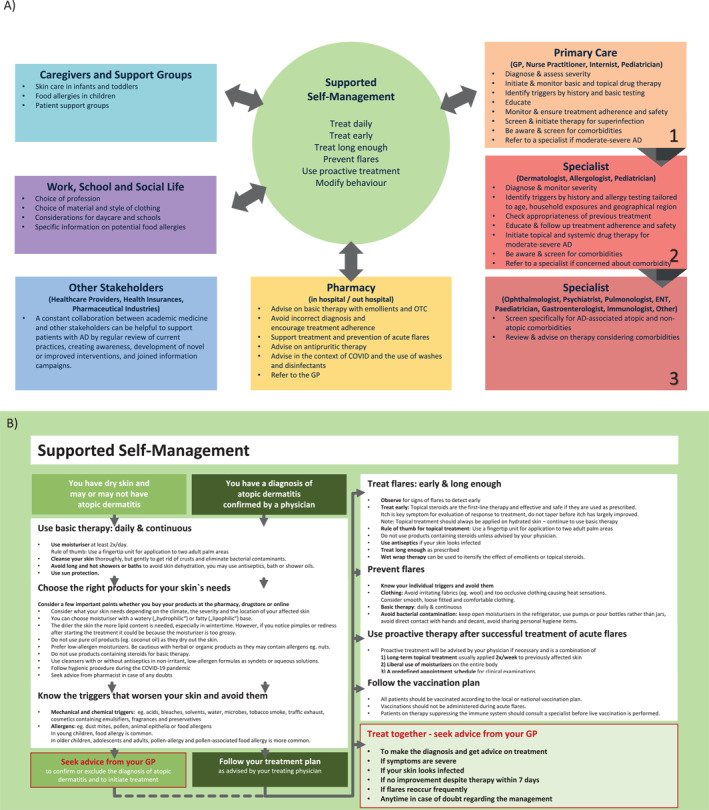

**Figure:**
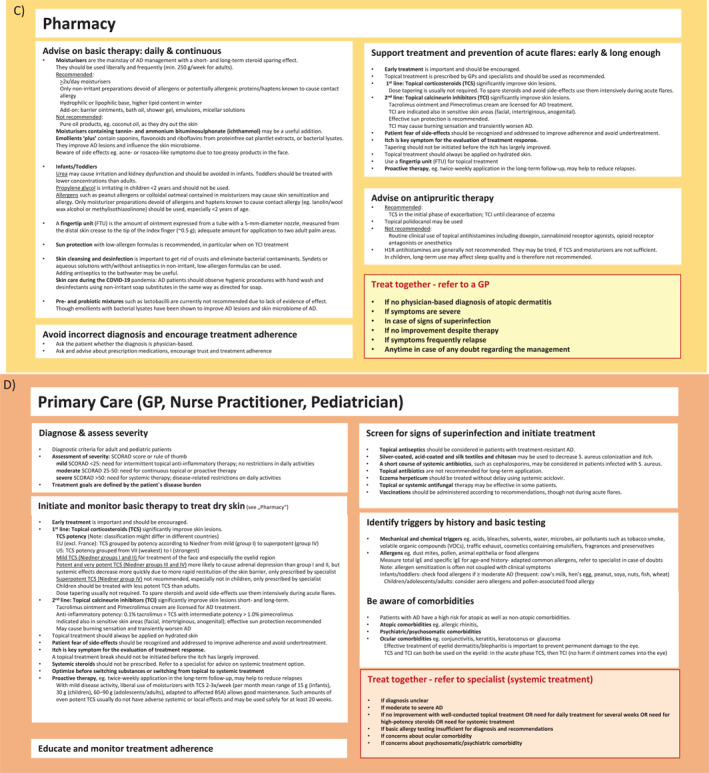

**Figure:**
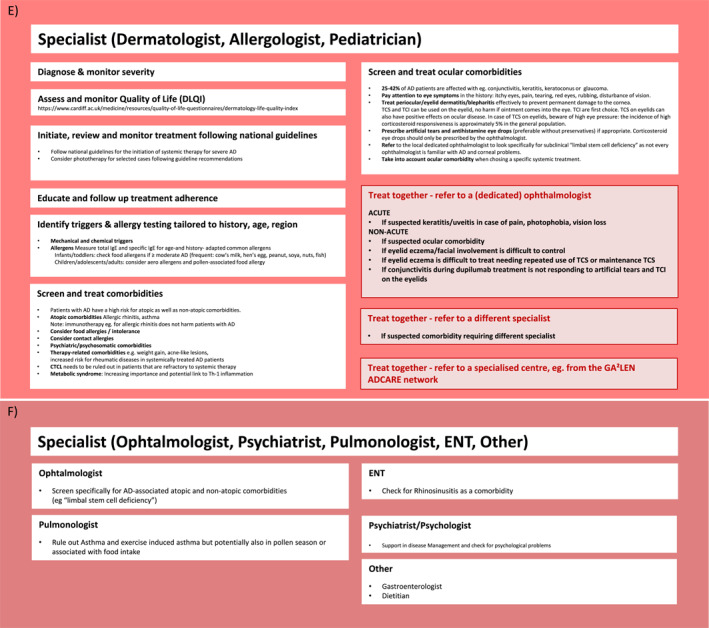

**Figure:**
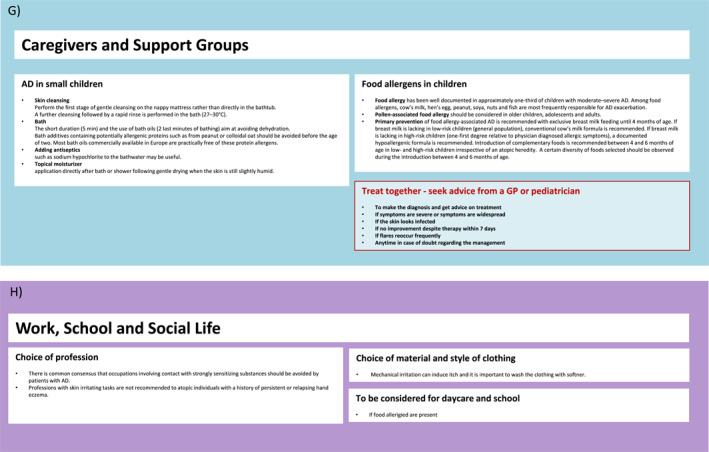

FIGURE 3ICP—additional information. (A) Supported self‐management.^20^, ^23^, ^34^, ^37^, ^38^ (B) Pharmacy.^38^ (C) Primary care I.^36^, ^37^ (D) Primary care II.^20^, ^23^, ^39^, ^40^ (E) Specialist (dermatologist, allergologist, pediatrician).^20^ ICPs, integrated care pathways.

**Figure:**
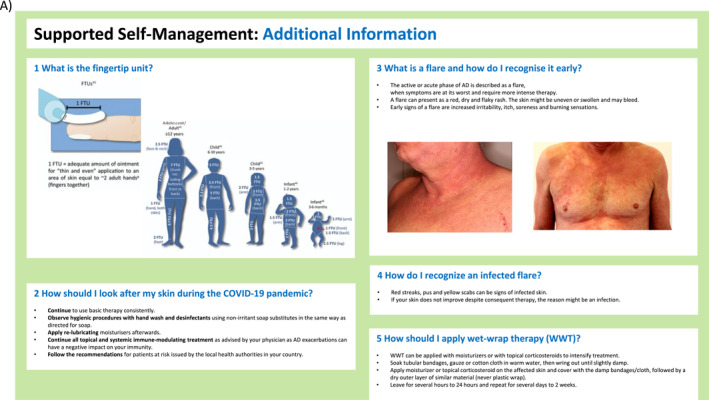

**Figure:**
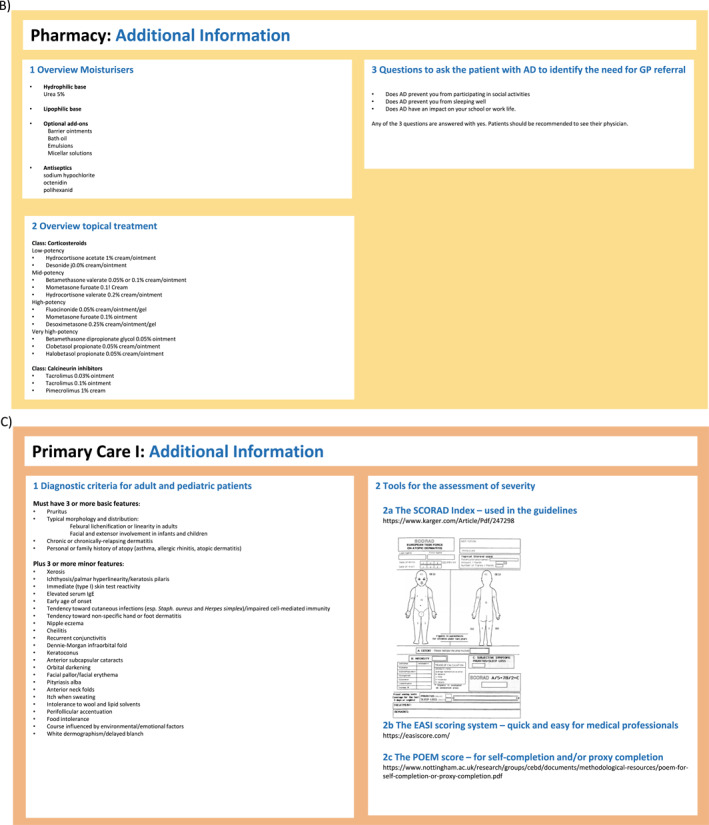

**Figure:**
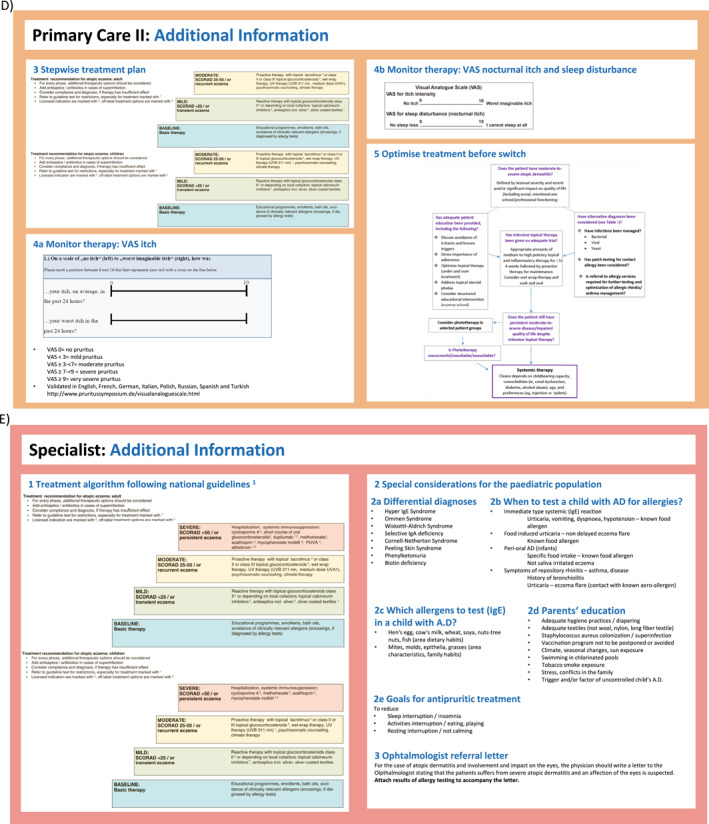

Adherence to AD therapy is often poor, particularly to adequate moisturiser application and to topical corticosteroid (TCS) treatment, the latter mainly due to the fear of side effects and steroid withdrawal symptoms.^42^, ^43^ GPs and primary care pediatricians are in a key position to improve compliance before specialist referral and the next treatment steps to more potent therapies are considered. Patients need to be educated to build up the competence and the confidence to consequently manage acute flares and long‐term maintenance treatment, which is known to effectively promote disease control.^44^

Patients with AD have a high risk of atopic as well as non‐atopic comorbidities (Figure 1A). For example, they have a significant and disease severity‐dependent increased risk of the development of ocular morbidities, which might affect 25%–42% of AD patients. These are often underdiagnosed and underrated—even by the specialists—and can in some cases result in permanent corneal damage.^45^, ^46^, ^47^ Awareness and early referral to a dedicated specialist in case of any suspicion is paramount.

Of equal importance and often overlooked are psychosomatic and psychiatric morbidities such as sleep disturbances in time and quality, anxiety, and depression.^48^ Effective screening should systematically be part of AD management and therefore has been included in the AD‐ICPs.

### 4.1 Diagnostics in AD—Should treatment targets be based on clinical scores, or on symptoms/QoL? What is feasible in a daily practice setting?

In the primary care setting, diagnosing, and staging of AD should be based on clinical criteria including the assessment of itching and sleep disturbance using visual analogue scales and overall quality of life. An adaptation of the diagnostic approach to the patients' age is required. The recommended diagnostic tool should be simple and could be supported by digital applications.

Differential diagnosis is important and potentially challenging, as for example, early stages of cutaneous T‐cell lymphoma might be misdiagnosed in patients with AD.^49^

Criteria for AD specialist referral (see Figure 1B) should be provided considering the distribution pattern (localised vs. widespread, extent and localisation of affected body surface area), the need for daily treatment for several weeks, the need for high potency steroids, the response to treatment, the recurrence of symptoms, infections, the presence of comorbidities, the age of disease onset, and the impact on quality of life.

Scores, such as SCORAD, EASI, POEM, or DLQI/cDLQI, should be reserved for specialists as training in using these scoring systems is crucial.

### 4.2 Comorbidities in AD

Atopic and non‐atopic comorbidities have a considerable impact on disease burden and treatment options and require a multidisciplinary, integrated concept for their prevention, early detection, monitoring, and treatment (Figure 1A). Early treatment considering all comorbidities might prevent the atopic march in selected individuals.^9^, ^10^, ^11^

For example, contact allergies in patients with AD require consideration when choosing the type of topical treatment. With regard to food allergies, it is important to distinguish between small children and adults, between immediate and delayed reactions, and between allergy and intolerance for a better description of the potential phenotype of AD that may have implications for different systemic therapies.

The knowledge on non‐atopic comorbidities in AD is increasing; however, the associations are not yet fully understood. For example, new data indicate autoimmune comorbidity in adults with AD, especially autoimmune dermatological, gastrointestinal and rheumatological diseases.^15^, ^16^ Furthermore, the potential link of the metabolic syndrome to Th‐1 inflammation should be addressed. At present, US and Asian data indicate cardiometabolic diseases with gender‐specific differences as a comorbidity of AD, while data from European patients do not support this.^14^, ^50^, ^51^, ^52^

As an example of multidisciplinary efforts, an Italian team created a patient questionnaire in order to detect type 2 inflammatory disorders and to guide subsequent multidisciplinary management.^53^

A holistic approach and an investment in integrated mental health services are required to address the higher risk for psychological stress, sleep, and mental health disorders, such as attention‐deficit/hyperactivity disorder, anxiety, depression, disorder of behaviour, autism, and suicide.

### 4.3 Rare but severe ocular comorbidities

Ophthalmological symptoms in patients with AD may be under detected and underestimated, all the more as patients can be asymptomatic.

To diagnose and treat ocular comorbidities in AD, ophthalmological symptoms should be detected by careful history taking of the patient. These could be itchy eyes, pain, tearing, red eyes, rubbing, and disturbance of vision.

To prevent ocular comorbidity, it is important to treat peri‐ocular/eyelid eczema adequately to prevent corneal damage. Topical calcineurin inhibitors (TCI) are the first choice of treatment. Use of TCS on the eyelids can have deleterious effects on the incidence of ocular disease. In this case, high eye pressure should be screened because high corticosteroid intraocular pressure responsiveness is approximately 5% in the general population.^54^

To treat conjunctivitis, dermatologists/allergologists can prescribe artificial tears and antihistamine/mast cell stabiliser eye drops (preferably without preservatives). Corticosteroid eye drops should preferably be prescribed by an ophthalmologist.

Patients should be referred promptly to an ophthalmologist in the case of pain, photophobia, or vision loss, all of which could indicate keratitis/uveitis. Patients should be referred non‐acutely (i) in case of moderate‐to‐severe AD and eyelid eczema/facial involvement, which is difficult to control, (ii) if eyelid eczema requires repeated use of TCS or maintenance TCS, or (iii) if conjunctivitis during biological treatment is not responding to artificial tears and TCI on the eyelids, or (iv) before starting a systemic therapy. As dedicated ophthalmologists are sparse, it is important to formulate precise referral criteria.

Ocular comorbidity should be one of the factors to consider when choosing a specific systemic treatment.^45^, ^46^, ^47^

### 4.4 Topical treatment—Health care provision: Needs and limitations

Dysbiosis and the spectrum of sensitisation, especially mold (fungi), are important in the contemporary concept of barrier impairment in AD and influence the choice of topical treatment.^55^, ^56^

Topical treatment encompasses the preventative and supportive use of baseline therapeutics such as emollients and emollients ‘plus’, the latter containing saponins, flavonoids, riboflavins or bacterial lysates. Even for basic treatment with emollients, high costs can be a problem depending on economic status. In some places, there are also local cheaper alternatives that could be used potentially. In India, for example, coconut oil is often used but patients should be advised that low‐quality substitutes may also have negative effects like contact allergy.

Topical treatment also includes anti‐inflammatory substances such as corticosteroids and calcineurin inhibitors. For topical steroids, it is recommended to use primarily the more modern substances like mometasone, which have a lower level of cutaneous adverse events and do not lead to systemic levels.^57^

The rationales, advantages, and limitations of treatment with topical therapies in AD are summarised in Table 1. Topical treatments are indicated as recommended by the guidelines and should be optimised before changing the type of therapy. The transition points from topical treatments have been laid out in the European guideline and in IEC recommendation papers.^24^, ^25^, ^39^, ^58^ However, it must be noted that treating large areas of the body with topical treatment has a severe negative impact on the quality of life and can influence the decision to move for systemic care.

**TABLE 1:** Rationale, needs and limitations for topical therapy in atopic dermatitis.

Rationale	To prevent and improve barrier disruptions To control inflammation To restore and maintain the skin microbiome To address specific problems such as itch, pain, superinfections, or exudation
Advantages	Connection with the problem of barrier dysfunction Efficiency when used adequately Control of side effects Flexible dosing and application modes Versatility in including further compounds General acceptance by most patients
Limitations	Burden of time No reimbursement from health insurances for basic therapy High costs for some of the newer topical treatments
Needs	Development of easy‐to‐use clinical scores to monitor treatment success as the validated scores such as SCORAD, EASI or DLQI are excellent in clinical trials but too time consuming for daily routine Further criteria should be developed for decision making in step up or down in treatment: The objective and subjective burden, the patient's history, time course of the disease, the patient's preferences and response to topical treatment

Access to drugs is mostly good but varies by country, for example, in some countries a limited use of TCI was noted. In addition, reimbursement systems differ between countries and can further limit effective treatment. Even if reimbursement is given, guideline compliance shows large gaps, mostly due to the physicians' prescribing patterns. Concerns from both, patients and physicians, about the adverse effects of corticosteroid can limit effective treatment. Regular training of primary care clinicians by specialists could help to close these gaps.

In conclusion, there is a clear differential need for topical treatment in AD with mostly preventive, curative, or supportive reasons. There is a consensus about the criteria for using topical versus systemic treatment. Optimising treatment before switching is key. However, the current health care quality of AD with topical treatments is diverse and the benefits of topical treatments in individual patients are highly variable.

### 4.5 Systemic treatments in AD

Most patients with mild‐to‐moderate AD respond adequately to optimised topical treatment and avoidance of exacerbating factors. The management of bacterial, viral and/or fungal skin infections is of major importance as they can be the cause of acute exacerbations of disease severity or resistance to treatment.

However, many patients may not have adequate disease control with topical treatment alone or in combination with phototherapy using UVB or UVA, which can be of value in selected cases. In these patients and those with moderate‐to‐severe AD, systemic therapy is needed to control skin inflammation. The decision when to start a systemic therapy can be difficult, given the known risks of traditional immunosuppressants, which may cause concern about infections, particularly during the COVID‐19 pandemic.^39^

The availability of systemic drugs is subject to country‐specific differences as well as payment and out‐of‐pocket costs in the private versus general health care sections.

Off label drugs still play a role in the management of AD. However, licensed therapies should be considered first. Several new therapies approved for the treatment of AD are available, such as biologicals (dupilumab and tralokinumab) and JAK inhibitors (baricitinib, upadacitinib and abrocitinib). Besides, further new therapies targeting other pathogenic mechanisms such as IL31 (nemolizumab) are emerging.^59^, ^60^, ^61^

The choice of systemic treatment in AD is dependent on the patients' age, efficacy, safety, comorbidities, and also the economic burden of the treatment (Table 2). Furthermore, specific concerns exist for some of the new agents and treating physicians should be familiar with the contraindications and appropriate laboratory follow up.

**TABLE 2:** Overview of currently licensed treatments for atopic dermatitis and/or other allergic comorbidities.

Drug	Licensed for atopic dermatitis	Licensed for allergic asthma	Licensed for chronic rhinosinusitis +/− nasal polyposis	Licensed for allergic rhinitis	Licensed for food allergies	Other
Dupilumab	X	X	X	‐	‐	‐
	Adults	Adults	Adults			
	Children ≥6 years	Children ≥6 years				
Tralokinumab	X	‐	‐	‐	‐	‐
	Adults					
Bariticinib	X	‐	‐	‐	‐	Adults with rheumatoid arthritis
	Adults					
Upadacitinib	X	‐	‐	‐	‐	Adults with rheumatoid arthritis, psoriatic arthritis, ankylosing spondylitis
	Adults					
	Children ≥12 years					
Abrocitinib	X	‐	‐	‐	‐	‐
	Adults					
Methotrexate	‐	‐	‐	‐	‐	Adults with rheumatoid arthritis, juvenile idiopathic arthritis, psoriasis, Crohn's disease
Cyclosporin A	X (>16 years in Germany and Austria)	‐	‐	‐	‐	Adults with rheumatoid arthritis, psoriasis, atopic keratoconjunctivitis

For certain patient subgroups, specific internationally published recommendations for systemic treatments exist, for example, for patients with comorbidities (e.g. asthma, rheumatoid arthritis), pregnancy, history of cancer, and planned vaccinations. Further subgroups require specific considerations, such as elderly patients and breastfeeding mothers.

Children and adolescents as a target group require special attention to safety aspects and for some substances specific adverse effects need to be considered, for example, for methotrexate. However, particularly in adolescents, the impact of AD on the quality of life is considerably high and may differ from other age groups.^62^ Furthermore, adequate disease control in children and adolescents may affect the allergy march.^63^ Fortunately, novel treatments, such as dupilumab, are now also available for pediatric patients.

### 4.6 New and emerging treatments: Biologics

The ideal aim is complete control by modern treatment achieving symptom‐free life while acknowledging that this cannot be easily achieved in a complex, chronic immune‐mediated inflammatory disease. In that case, there must be a joint discussion between the physician and patient about what is the realistic treatment aim, for example, aiming for symptom‐free subthreshold eczema as well as the avoidance of flare ups.

Applications of newly developed drugs in clinical studies or already in daily practice show substantial progress in the treatment of moderately‐to‐severely affected patients with AD not responsive to standard topical treatments with corticosteroids or calcineurin inhibitors alone. Moreover, novel treatment approaches generate new knowledge about the (anti)inflammatory effects of immune modulation in AD and the heterogeneity of patient subgroups, which may stimulate further innovations in this field.

When evaluating new therapies for AD, special importance is attached to the assessment of patient‐related outcomes. For the future, drugs highly effective for symptoms such as pruritus with a good benefit/risk ratio, with the possibility of individual dosing, and a rapid effect on symptom improvement after initiation of therapy are desired.

Dupilumab was the first specific therapeutic monoclonal antibody approved for the treatment of AD in 2017. The AD TREAT Germany registry demonstrated that dupilumab to date is by far the most prescribed systemic drug for AD with proven efficacy.^41^, ^60^, ^64^, ^65^ Since autumn 2020, three JAK inhibitors have been approved for the treatment of AD in Europe (baricitinib, upadacitinib and most recently abrocitinib).^61^, ^66^ Moreover, the anti‐IL‐13 specific monoclonal antibody tralokinumab was approved for the treatment of AD in adults.^67^ While dupilumab is approved for adults as well as children, the benefits and safety of monoclonal antibodies and JAK inhibitors in moderate‐to‐severe AD in children and adolescents are still under investigation.^68^

Although anti‐IgE treatment is effective in some patients with asthma, it has not been proven to be effective in patients with AD so far.^69^, ^70^

The efficacy of house dust mite (HDM) sublingual immunotherapy (SLIT) has been shown in patients with airway allergic diseases. Over the last years, several randomised controlled studies demonstrated that HDM SLIT represents an additional therapeutic tool for the treatment of mild‐to‐moderate AD in selected patients with comorbid‐allergic rhinitis and/or asthma.^71^, ^72^

So far, it is still unclear what patients benefit most from which systemic therapy. The guidance on the treatment approach in patients with moderate‐to‐severe AD and those with comorbidities is an important future task. Many of the newly licensed as well as emerging treatments have a positive effect not only on AD but also on atopic and non‐atopic comorbidities.

Table 2 gives an overview of the currently licensed treatments for AD and/or other allergic comorbidities.

Short comparative trials are available; however, most treatments have not been compared head‐to‐head yet.^59^ In acknowledgment of the rapidly increasing evidence for a variety of emerging new therapies, an international group of clinicians, scientists and patients has conducted a living systematic review and network meta‐analysis to provide relative efficacy, safety and impact on quality of life for available treatments. The results are updated regularly and made easily accessible on the website www.eczematherapies.com for patients and clinicians.^73^

At present, no treat‐to‐target framework exists to guide the optimal use of systemic therapies in AD. As the current evidence‐base for specific recommendations is still sparse, an international consensus framework was sought based on expert opinion and informed by extensive clinical experience. A clinical algorithm has been proposed to guide shared decision‐making for systemic treatment, continuation, modification, or discontinuation in adults with moderate‐to‐severe AD. This work is intended to be a starting point and foundation to inform and stimulate a wider debate.^58^

### 4.7 AD in the pediatric population

Cohort studies demonstrate a cumulative incidence of 22.8% in children aged 0–6 years and an AD lifetime prevalence of 21.3% in adolescents and 34.1% in adults.^3^, ^74^, ^75^

Special considerations in the pediatric population concern the complex interrelations between an evolving disease with different phenotypes and endotypes, a developing child, the maturation of skin, the immune system, and metabolism.

A correct diagnosis considering frequent and rare differential diagnoses needs to be ensured by a specialist when needed. Food allergies and immunodeficiencies must be considered in patients with severe and/or persistent courses, particularly in patients with an early onset. Children with severe and/or therapy‐resistant AD in combination with high IgE levels and/or hypereosinophilia should be investigated for genetic inborn errors of immunity with the expertise of allergologists, immunologists, and geneticists.^76^

Furthermore, parents' perceptions should be considered, treatment safety assessed by age, long‐term disease control achieved, and comorbidities recognised early and prevented when possible. It is important to look at the complex interaction the disease has both on the family life as well as its impact on the life of the child itself when for instance at school.

Basic skin care and topical treatment should be chosen considering the safety, tolerability, hypoallergenic properties, and parent and child's acceptance. Pruritus control is an important therapy goal. A personalised written action plan and team‐school training to build self‐confidence and support self‐management should be provided for patients and caregivers.^20^, ^23^

Skin‐barrier dysfunction can be apparent in the first weeks of life before the development of AD, suggesting that interventions to improve skin barrier function from infancy have the potential to prevent the skin condition.^77^ Gentle skin care for newborns supports skin function and ongoing postnatal skin maturation. Hence, recommendations on bathing and skin moisturisation, umbilical cord and diaper area care, and sun protection should be given to parents by midwives, nurses, and physicians.^78^ However, a Cochrane meta‐analysis concluded that skin care interventions such as emollients during the first year of life in healthy infants are probably not effective in preventing AD. Further work is needed to understand whether different approaches to infant skin care might promote or prevent AD.^79^, ^80^, ^81^

### 4.8 Living with the disease

AD is a chronic disease with different grades of severity, and it needs a reassessment of diagnostic features since new triggers may evolve, and an adaptation of the therapy may be needed. Ideally, depending on the grade or severity of AD, a physician should be in regular contact with the patient. The patient should also be empowered as much as possible to treat their own disease according to the fluctuating needs or disease status, but at the same time is trained enough at regular visits to assess these changes and triggers sensibly. This also includes early advising of parents about the prognosis of the disease and allergy prevention, for example, not smoking and early disease detection.

### 4.9 The role of the pharmacist

Pharmacists should play a role in improving the patient's adherence with therapy, to reinforce and emphasise physicians' messages, and to prevent prescription mistakes (e.g. misdosing, incorrect drug name, individual patient contraindications) and drug‐drug‐interactions.

Currently, pharmacists mainly counsel on moisturisers and over the counter medications. The training of pharmacists is crucial considering the‐phobia of patients and pharmacists. Specifically trained dermatological ‘speciality’ pharmacists within a pharmacy team would be desirable, providing the support outlined in Figure 2C. The geographical variability of the resources and training will significantly affect the role the pharmacists play in AD treatment. In some countries, the role of an even highly specialised pharmacist in the context of systemic therapies is an important development.

## 5 AN OUTLOOK FOR AD

While the AD‐ICPs focus on the current situation, this concluding chapter aims at the future perspectives with potential implications for research activities.

### 5.1 Limited resources

AD is increasing in prevalence in lower‐ and middle‐income countries of Asia, Africa, Latin America, and the Middle East. Challenges include cost, access to care, and lack of specialists. Furthermore, most of the available diagnostic criteria and treatment guidelines are based on European and North American populations and only few trials report the ethnicity of the study population.^82^ Although AD presents similarly across racial and ethnic groups, some features may be different in patients with darker skin, as well as drug pharmacokinetics and adverse effects in different ethnicities are yet to be investigated. The unmet medical need for the management of AD in developing countries can be addressed by the training of specialists, improvement of access to and affordability of care.^83^, ^84^ Furthermore, more financial support is needed for educational programs. This can save costs as well‐educated patients can more easily control their disease.

### 5.2 A personalised approach for AD patients

Despite its complex pathophysiology and variable clinical phenotype, AD is often considered a single disease and treated with a uniform approach. More tailored prevention and therapeutic strategies are being explored, such as by the BIOMAP Consortium, aiming to stratify AD patients according to their phenotype and endotype with the support of new biomarkers.^85^, ^86^ Such a personalised approach may help to assign available and newly emerging drugs to those patients with the best benefit/risk ratio.^22^ Associations found using machine learning to perform deep phenotyping and identification of severity‐associated factors might contribute to improving the monitoring of predisposed patients, and personalised disease management.^87^

### 5.3 Recent geopolitical developments, the impact of climate change on allergies and AD

Addressing climate change may be the greatest challenge and most impactful intervention for global health in the 21st century; at the same time, failure to do so could destroy all the progress that has been made in public health in recent decades.^88^, ^89^, ^90^ With regard to allergies, climate change affects the severity of symptoms and increases in incidence and prevalence through its impact on pollen counts: The pollen season is getting longer, pollen is more allergenic and new pollen such as ragweed are becoming native to Europe.^91^, ^92^, ^93^ Patients suffering from AD triggered by pollen are more likely to be affected by these consequences of climate change. Furthermore, increasing heat waves are likely to be a trigger for more eczema exacerbations. Of note, both low and high ambient temperatures can increase the risk of outpatient visits.^94^ More exposure‐response association analyses are needed to understand the effects of ambient temperature and eczema to develop preventive measures. Furthermore, no data are available on the effect of heat on local and systemic treatment of atopic eczema.

### 5.4 ICPs by digital harmonisation

As part of a patient‐centred approach, digitally supported ICPs could shorten the time to diagnosis, guide the patient in implementing the stepwise treatment plan and collect feedback from patients. They could also facilitate shared decision making between specialists. The existing MASK air app is the optimal app to add to comorbidities and AD. As a future perspective, this app should be embedded in other disease management systems. In the near‐term future, digital health solutions could be provided at the government level in the national and other frequent languages for nomadic working citizens.

## AUTHOR CONTRIBUTIONS

**Torsten Zuberbier**: Conceptualization (equal); data curation (equal); formal analysis (equal); investigation (equal); methodology (equal); project administration (equal); resources (equal); supervision (equal); visualization (equal); writing – review & editing (equal). **Amir Abdul Latiff**: Formal analysis (equal); methodology (equal); writing – review & editing (equal). **Xenofon Aggelidis**: Conceptualization (equal); data curation (equal); writing – review & editing (equal). **Matthias Augustin**: Conceptualization (equal); formal analysis (equal); writing – review & editing (equal). **Radu‐Gheorghe Balan**: Conceptualization (equal); formal analysis (equal); writing – review & editing (equal). **Christine Bangert**: Conceptualization (equal); formal analysis (equal); writing – review & editing (equal). **Lisa Beck**: Conceptualization (equal); formal analysis (equal); writing – review & editing (equal). **Thomas Bieber**: Formal analysis (equal); writing – review & editing (equal). **Jonathan A. Bernstein**: Formal analysis (equal); writing – review & editing (equal). **Marta Bertolin Colilla**: Formal analysis (equal); writing – review & editing (equal). **Alejandro Berardi**: Formal analysis (equal); writing – review & editing (equal). **Anna Bedbrook**: Formal analysis (equal); writing – review & editing (equal). **Carsten Bindslev‐Jensen**: Formal analysis (equal); writing – review & editing (equal). **Jean Bousquet**: Formal analysis (equal); writing – review & editing (equal). **Marjolein de Bruin‐Weller**: Formal analysis (equal); writing – review & editing (equal). **Dayanne Bruscky**: Formal analysis (equal); writing – review & editing (equal). **Betul Buyuktiryaki**: Formal analysis (equal); writing – review & editing (equal). **Giorgio Walter Canonica**: Formal analysis (equal); writing – review & editing (equal). **Carla Castro**: Formal analysis (equal); writing – review & editing (equal). **Natia Chanturidze**: Formal analysis (equal); writing – review & editing (equal). **Herberto Jose Chong‐Neto**: Formal analysis (equal); writing – review & editing (equal). **Chia‐Yu Chu**: Formal analysis (equal); writing – review & editing (equal). **Leena Chularojanamontri**: Formal analysis (equal); writing – review & editing (equal). **Michael Cork**: Conceptualization (equal); formal analysis (equal); writing – review & editing (equal). **Roberta F. J. Criado**: Formal analysis (equal); writing – review & editing (equal). **Laia Curto Barredo**: Funding acquisition (equal); writing – review & editing (equal). **Adnan Custovic**: Formal analysis (equal); writing – review & editing (equal). **Ulf Darsow**: Formal analysis (equal); writing – review & editing (equal). **Arben Emurlai**: Formal analysis (equal); writing – review & editing (equal). **Ana de Pablo**: Formal analysis (equal); writing – review & editing (equal). **Stefano Del Giacco**: Formal analysis (equal); writing – review & editing (equal). **Giampiero Girolomoni**: Formal analysis (equal); writing – review & editing (equal). **Tanja Deleva Jovanova**: Formal analysis (equal); writing – review & editing (equal). **Mette Deleuran**: Formal analysis (equal); writing – review & editing (equal). **Nikolaos Douladiris**: Formal analysis (equal); writing – review & editing (equal). **Bruno Duarte**: Formal analysis (equal); writing – review & editing (equal). **Ruta Dubakiene**: Formal analysis (equal); writing – review & editing (equal). **Esben Eller**: Formal analysis (equal); writing – review & editing (equal). **Batya Engel‐Yeger**: Formal analysis (equal); writing – review & editing (equal). **Luis Felipe Ensina**: Conceptualization (equal); formal analysis (equal); writing – review & editing (equal). **Nelson Rosario Filho**: Formal analysis (equal); writing – review & editing (equal). **Carsten Flohr**: Formal analysis (equal); writing – review & editing (equal). **Daria Fomina**: Formal analysis (equal); writing – review & editing (equal). **Wojciech Francuzik**: Formal analysis (equal); writing – review & editing (equal). **Maria Laura Galimberti**: Formal analysis (equal); writing – review & editing (equal). **Ana M. Giménez‐Arnau**: Formal analysis (equal); writing – review & editing (equal). **Kiran Godse**: Formal analysis (equal); writing – review & editing (equal). **Charlotte Gotthard Mortz**: Formal analysis (equal); writing – review & editing (equal). **Maia Gotua**: Formal analysis (equal); writing – review & editing (equal). **Michihiro Hide**: Formal analysis (equal); writing – review & editing (equal). **Wolfram Hoetzenecker**: Formal analysis (equal); writing – review & editing (equal). **Nicolas Hunzelmann**: Formal analysis (equal); writing – review & editing (equal). **Alan Irvine**: Formal analysis (equal); writing – review & editing (equal). **Carolyn Jack**: Formal analysis (equal); writing – review & editing (equal). **Ioanna Kanavarou**: Formal analysis (equal); writing – review & editing (equal). **Norito Katoh**: Funding acquisition (equal); writing – review & editing (equal). **Tamar Kinaciyan**: Formal analysis (equal); writing – review & editing (equal). **Emek Kocatürk**: Formal analysis (equal); writing – review & editing (equal). **Kanokvalai Kulthanan**: Formal analysis (equal); writing – review & editing (equal). **Hilde Lapeere**: Formal analysis (equal); writing – review & editing (equal). **Susanne Lau**: Formal analysis (equal); writing – review & editing (equal). **Mariana Machado Forti Nastri**: Formal analysis (equal); writing – review & editing (equal). **Michael Makris**: Formal analysis (equal); writing – review & editing (equal). **Eli Mansour**: Formal analysis (equal); writing – review & editing (equal). **Alexander Marsland**: Formal analysis (equal); writing – review & editing (equal). **Mara Morelo Rocha Felix**: Formal analysis (equal); writing – review & editing (equal). **Ana Paula Moschione Castro**: Formal analysis (equal); writing – review & editing (equal). **Eustachio Nettis**: Formal analysis (equal); writing – review & editing (equal). **J. F. Nicolas**: Formal analysis (equal); writing – review & editing (equal). **Audrey Nosbaum**: Formal analysis (equal); writing – review & editing (equal). **Mikaela Odemyr**: Formal analysis (equal); writing – review & editing (equal). **Niki Papapostolou**: Formal analysis (equal); writing – review & editing (equal). **Claudio A. S. Parisi**: Formal analysis (equal); writing – review & editing (equal). **Sushil Paudel**: Formal analysis (equal); writing – review & editing (equal). **Jonny Peter**: Formal analysis (equal); writing – review & editing (equal). **Prakash Pokharel**: Formal analysis (equal); writing – review & editing (equal). **Luis Puig**: Formal analysis (equal); writing – review & editing (equal). **Tamara Quint**: Formal analysis (equal); writing – review & editing (equal). **German Dario Ramon**: Formal analysis (equal); writing – review & editing (equal). **Frederico Regateiro**: Formal analysis (equal); writing – review & editing (equal). **Giampaolo Ricci**: Formal analysis (equal); writing – review & editing (equal). **Cristine Rosario**: Conceptualization (equal); formal analysis (equal); writing – review & editing (equal). **Cansin Sackesen**: Formal analysis (equal); writing – review & editing (equal). **Peter Schmid‐Grendelmeier**: Formal analysis (equal); writing – review & editing (equal). **Esther Serra‐Baldrich**: Formal analysis (equal); writing – review & editing (equal). **Kristina Siemens**: Conceptualization (equal); writing – original draft (equal); writing – review & editing (equal). **Cathrine Smith**: Formal analysis (equal); writing – review & editing (equal). **Petra Staubach**: Formal analysis (equal); writing – review & editing (equal). **Katarina Stevanovic**: Conceptualization (equal); project administration (equal); visualization (equal); writing – original draft (equal); writing – review & editing (equal). **Özlem Su‐Kücük**: Formal analysis (equal); writing – review & editing (equal). **Gordon Sussman**: Formal analysis (equal); writing – review & editing (equal). **Simona Tavecchio**: Formal analysis (equal); writing – review & editing (equal). **Natasa Teovska Mitrevska**: Formal analysis (equal); writing – review & editing (equal). **Diamant Thaci**: Formal analysis (equal); writing – review & editing (equal). **Elias Toubi**: Formal analysis (equal); writing – review & editing (equal). **Claudia Traidl‐Hoffmann**: Formal analysis (equal); writing – review & editing (equal). **Regina Treudler**: Formal analysis (equal); writing – review & editing (equal). **Zahava Vadasz**: Formal analysis (equal); writing – review & editing (equal). **Ingrid van Hofman**: Project administration (equal). **Maria Teresa Ventura**: Formal analysis (equal); writing – review & editing (equal). **Zhao Wang**: Formal analysis (equal); writing – review & editing (equal). **Thomas Werfel**: Formal analysis (equal); writing – review & editing (equal). **Andreas Wollenberg**: Formal analysis (equal); writing – review & editing (equal). **Ariana Yang**: Formal analysis (equal); writing – review & editing (equal). **Yik Weng Yew**: Formal analysis (equal); writing – review & editing (equal). **Zuotao Zhao**: Formal analysis (equal); writing – review & editing (equal). **Ricardo Zwiener**: Formal analysis (equal); writing – review & editing (equal). **Margitta Worm**: Formal analysis (equal); writing – review & editing (equal).

## CONFLICT OF INTEREST STATEMENT

T. Zuberbier has received institutional funding for research and/or honoria for lectures and/or consulting from Amgen, AstraZeneca, AbbVie, ALK, Almirall, Astellas, Bayer Health Care, Bencard, Berlin Chemie, FAES, HAL, Henkel, Kryolan, Leti, L'Oreal, Meda, Menarini, Merck, MSD, Novartis, Pfizer, Sanofi, Stallergenes, Takeda, Teva and UCB, Uriach; in addition, he is a member of ARIA/WHO, DGAKI, ECARF, GA^2^LEN and WAO. A. A. Latiff declares no COI. X. Aggelidis declares no COI. M. Augustin has received institutional funding for research and/or honoria for lectures and/or consulting and/or was a member of an advisory board from AbbVie, Almirall, Beiersdorf, Eli Lilly, Galderma, LEO, Pfizer and Sanofi‐Genzyme. R.‐G. Balan declares no COI. C. Bangert declares no COI. L. Beck declares no COI. T. Bieber was speaker and/or consultant and/or Investigator for AbbVie, Affibody, Almirall, Amagma, AnaptysBio, AOBiom, Arena, Aristea, Asana Biosciences, ASLAN pharma, Bayer Health, BioVerSys, Böhringer‐Ingelheim, Bristol‐Myers Squibb, Connect Pharma, Daichi‐Sanyko, Dermavant, DIECE Therapeutics, Domain Therapeutics, DS Pharma, EQRx, Galderma, Galapagos, Glenmark, GSK, Incyte, Innovaderm, IQVIA, Janssen, Kirin, Kymab, LEO, LG Chem, Lilly, L'Oréal, MSD, Medac, Nektar, Novartis, Numab, OM‐Pharma, Pfizer, Pierre Fabre, Q32bio, RAPT, Sanofi/Regeneron, UCB, Union Therapeutics. He is the founder and chairman of the board of the non‐profit biotech ‘Davos Biosciences’; in addition is a member of the advisory board for Sanofi and Novartis, president of the Erich‐Hoffmann society in Bonn, member of the scientific board of CK‐CARE, chair of the board of directors of CK‐CARE, chair of the board of directors of Davos Biosciences (non‐profit company), member of the board of directors Medicine Campus Davos. J. A. Bernstein has received institutional funding for research and/or honoria for lectures and/or consulting from Allakos, Sanofi‐Regeneron, AZ, Novartis, Genentech, Celldex, TEVA; in addition is the AAAAI president, is on the board of advisory for WAO, Inerasma, and AFI chairperson. M. B. Collila declares no COI. A. Berardi declares no COI. A. Bedbrook declares no COI. C. Bindslev‐Jensen declares no COI. J. Bousquet reports personal fees from Cipla, Menarini, Mylan, Novartis, Purina, Sanofi‐Aventis, Teva, Uriach, other from KYomed‐Innov, other from Mask‐air‐SAS, outside the submitted work. M. de Bruin‐Weller reports grants and personal fees from Abbvie, personal fees from Almirall, personal fees from Aslan, grants and personal fees from Eli Lilly, personal fees from Galderma, personal fees from Janssen, grants and personal fees from Leo Pharma, grants and personal fees from Pfizer, grants and personal fees from Regeneron/Sanofi, outside the submitted work. D. Bruscky declares no COI. B. Buyuktiryaki declares no COI. G. W. Canonica reports personal fees from Ðanofi, personal fees from Stallergenes, personal fees from Genzyme, personal fees from Menarini, personal fees from GSK, personal fees from Chiesi, outside the submitted work. C. Castro has received institutional funding for research and/or honoria for lectures and/or consulting from Pfizer, Abbvie, Sanofi, Jannsen, L’Oreal, Eucerin, and Galderma and plays a leadership role in Atopic Dermatitis Group SAD. N. Chanturidze declares no COI. H. J. Chong‐Neto declares no COI. C.‐Y. Chu reports personal grants, fees and/or other from AbbVie, Lilly, Novartis, Oneness Biotech, Pfizer, Regeneron, Roche, Sanofi, United BioPharma Viatris, outside the submitted work. L. Chularojanamontri has received grants/research support from Novartis. M. Cork reports grants and personal fees from Hyphens Pharma, grants and personal fees from Johnson & Johnson, grants and personal fees from Pfizer, grants and personal fees from Sanofi, grants and personal fees from L'Oreal, grants and personal fees from Leo Pharma, grants and personal fees from Regeneron, personal fees from Procter & Gamble, personal fees from UCB, outside the submitted work; and is a voluntary medical adviser to the National Eczema Society, UK. R. F. J. Criado has received institutional funding for research and/or honoria for lectures and/or consulting and/or was a member of an advisory board from Takeda, Novartis, Sanofi, Pfizer, Abbvie, and Lilly. L. Curto Barredo reports personal fees and non‐financial support from Sanofi, Leo Pharma, Abbvie, and Lilly, outside the submitted work. A. Custovic reports personal fees from Novartis, Sanofi, Stallergenes Greer, AstraZeneca Worg Pharmaceuticals, and GSK, outside the submitted work. U. Darsow declares no COI. A. Emurlai declares no COI. A. de Pablo declares no COI. S. Del Giacco reports grants and personal fees from Sanofi, outside the submitted work. G. Girolomoni declares no COI. T. Deleva Jovanov declares no COI. M. Deleuran has received institutional funding for research and/or honoria for lectures and/or consulting and/or was a member of an advisory board from Leo Pharma, Abbvie, Eli‐Lilly, Regeneron, Sanofi Genzyme, Pfizer, La Roche Posay, Pierre Farbe, Novartis, Almirall, Arena Pharmaceuticals, ASLAN Pharmaceuticals, Incyte and Kymab. N. Douladiris declares no COI. B. Duarte has received honoraria as a speaker from Sanofi, Abbvie, Leo Pharma, and Lilly. R. Dubakiene declares no COI. E. Eller declares no COI. B. Engel‐Yeger declares no COI. L. F. Ensina reports personal fees from NOVARTIS, non‐financial support from SANOFI, and personal fees from ABBVIE, outside the submitted work. N. Rosario Filho received honoraria as speaker and consultant funded research grant for Sanofi, Abbvie, AstraZeneca, Boehringer, Chiesi, Novartis, Mantecorp, Janssen, Vertex, Abbott. C. Flohr declares no COI. D. Fomina declares no COI. W. Francuzik declares no COI. M. L. Galimberti reports personal fees from Janssen and Novartis. A. Giménez‐Arnau has received institutional funding for research and/or honoria for lectures and/or consulting from Almirall, Amgen, Astra Zeneca, Avene, Celldex, ESXCIENT, Instituto Carlos III‐FEDER, Menarini, Novartis, Sanofi‐Regeneron, Thermo Fisher, and Uriach Pharma/Neucor. K. Godse declares no COI. C. G. Mortz declares no COI. M. Gotua declares no COI. M. Hide reports grants and personal fees from Novartis, grants and personal fees from Sanofi, grants and personal fees from Kyowa‐Hakko‐Kirin, grants and personal fees from Mitsubishi‐Tanabe, and grants and personal fees from Uriach, outside the submitted work. W. Hoetzenecker declares no COI. N. Hunzelmann has received institutional funding for research and/or honoria for lectures and/or consulting from Abbvie, Leo Pharma, and Sanofi. A. Irvine has received institutional funding for research and/or honoria for lectures and/or consulting and/or was a member of an advisory board from Almirall, Abbvie, Eli Lilly, Pfizer, Benevolent AI, Arena, Novartis, Regeneron, Sanofi, Leo Pharma, Janssen, OM Pharma, has a pending patent with J and J, and is the president elect of the International Eczema Council. C. Jack reports grants from Innovaderm Research, McGill University Department of Medicine, MITACS, Canadian Dermatology Foundation, and Eczema Society of Canada, as well as grants, involvement in clinical studies, and/or consultancy work for Sanofi, Eli Lilly, AbbVie, Novartis, Valeant, Bausch, Pfizer, Amgen, Celgene, Janssen, Boehringer Ingelheim, Asana, LEO, Dermavant, AntibioTx, Neokera, Kiniksa, Ralexar, Arcutis, BMS, Boston, Cara, Concert, Incyte, Sienna, Aristea, Target PharmaSolution, and UCB. I. Kanavarou declares no COI. N. Katoh has received honoraria as a speaker/consultant for Sanofi, Maruho, Abbvie, Ely‐Lilly Japan, Mitsubishi Tanabe Pharma, Jansen Pharma, Taiho Pharmaceutical, Torii Pharmaceutical, Kyowa Kirin, Celgene Japan and Leo Pharma and has received grants as an investigator from Maruho, Ely‐Lilly Japan, Sun Pharma, Taiho Pharmaceutical, Torii Pharmaceutical, Boehringer Ingelheim Japan, Kyowa Kirin, Jansen Pharma, Boehringer Ingelheim Japan, A2 Healthcare, Abbvie, and Leo Pharma. T. Kinaciyan reports personal fees and other from BioCryst, grants, personal fees and other from Takeda, other from KalVista, personal fees from Novartis, personal fees from Hal Allergy, outside the submitted work. E. Kocatürk declares no COI. K. Kulthanan has received grants/research support from Novartis, honoraria/consultation fees from Novartis, Sanofi, A. Menarini, and Takeda. H. Lapeere has received institutional funding for research and/or honoria for lectures and/or consulting from Abbvie, Leo Pharma, Sanofi, Pfizer, and Almirall. S. Lau has received institutional funding for research and/or honoria for lectures and/or consulting from Sanofi, GSK, Leo Pharma, DBV, Allergopharma, Bencard, and Leti. M. M. F. Nastri declares no COI. M. Makris declares no COI. E. Mansour declares no COI. A. Marsland reports personal fees or other from Almirall, Galderma, Lilly, La roche Posay, Novartis, outside submitted work. M. Morelo Rocha Felix declares no COI. A. P. Moschione Castro reports being on the ABBVIE advisory board and Sanofi advisory board. E. Nettis reports personal fees from Sanofi, Leo Pharma, Chiesi, and Novartis. J. F. Nicolas declares no COI. A. Nosbaum declares no COI. M. Odemyr declares no COI. N. Papapostolou declares no COI. C. A. S. Parisi declares no COI. S. Paudel declares no COI. J. Peter has received honararia, travel support and/or educational grant funding from Novartis, Sanofi, AstraZeneca and Johnson and Johnson. P. Pokharel declares no COI. L. Puig reports grants and personal fees from AbbVie, grants and personal fees from Almirall, grants and personal fees from Amgen, grants and personal fees from Boehringer Ingelheim, grants and personal fees from Leo‐Pharma, personal fees from Bristol Myers Squibb, grants and personal fees from Lilly, grants and personal fees from Novartis, grants and personal fees from Pfizer, personal fees from Sandoz, grants and personal fees from Sanofi, grants and personal fees from UCB, during the conduct of the study. T. Quint declares no COI. G. D. Ramon declares no COI. F. Regateiro reports personal fees from Sanofi, Abbvie, Lilly and LEO Pharma, outside the submitted work. G. Ricci reports personal fees from Glasosmithkline and Recordiati and is a member of the Scientific Committee of the Italian Pediatric Dermatology Society. C. Rosario declares no COI. C. Sackesen declares no COI. P. Schmid‐Grendelmeier has received research grants from Christine Kühner Center for Allergy Research and Education CK‐CARE, has received personal fees from AbbVIe, Almiral, Galderma, LEO, Lilly, LÒreal, Novartis Pfizer, Pierre Favre, Sanofi‐Regeron, is on the advisory board for AbbVIe, Almiral, Galderma, LEO, Lilly, Pfizer, Sanofi‐Regeron, is a treasurer for the International Society for Atopic Dermatitis ISAC, chair in the Atopic Dermatitis Group in WAO, is a board member of the Swiss Patient Organization at the AHA Swiss Center for Allergy. E. Serra‐Baldrich has received personal fee payments and travel support from Abbvie, Lilly, Sanofi, Novartis, Pfizer, Galderma, and Leo Pharma. K. Siemens has received payment from the GA^2^LEN ADCARE Network for support of the present manuscript. C. Smith reports receiving a grant from the European Commission‐IMI. P. Staubach declares no COI. K. Stevanovic reports receiving a stipend from GA^2^LEN. Ö. Su‐Kücük declares no COI. G. Sussman has received research support from Aimmune, Amgen, Astra‐Zeneca, DBV technologies, Genentech, Kedrion S.p.A, Leo Pharma, Novartis, Sanofi, Regeneron, and ALK; and is a medical advisor and/or has received payment for lectures from Novartis, CSL Behring, Pfizer, Abbvie, Astra‐Zeneca, Nuvo Pharmaceuticals, and the Allergy Asthma and Immunology Society of Ontario. S. Tavecchio declares personal fee payments and travel cost payments from Sanofi, Abbvie, and Leo Pharma. N. Teovska Mitrevska declares no COI. D. Thaci reports grants and personal fees from AbbVie, personal fees from Almirall, personal fees from Bristol‐Myers Squibb, personal fees from Amgen, personal fees from Janssen, grants and personal fees from Leo‐Pharma, personal fees from Lilly, grants and personal fees from Novartis, personal fees from Pfizer, personal fees from Regeneron, personal fees from Sanofi, personal fees from Target, personal fees from UCB, during the conduct of the study. E. Toubi declares no COI. C. Traidl‐Hoffmann declares no COI. R. Treudler reports personal fees and grants from Sanofi, AbbVie, Pfizer, Lilly, and Novartis, outside submitted work. Z. Vadasz declares no COI. I. van Hofman declares no COI. M. T. Ventura declares no COI. Z. Wang declares no COI. T. Werfel reports personal fees and grants from Beiersdorf, Novartis, Leo Pharma, Abbvie, Janssen, Celgene, Galderma, Lily, and Sanofi, is on the advisory board for Abbvie, Janssen, Galderma, LEO, Lilly, Pfizer, Sanofi‐Genzyme, and Novartis, and is a board member for ETFAD, EAACI, ESDR, DGAKI, DDG. A. Wollenberg reports personal fees from AbbVie, Chugai, Galderma, LEO Pharma, Lilly, MedImmune, Novartis, Pfizer, Regeneron and Sanofi‐Aventis, and grants from LEO Pharma outside the submitted work. A. Yang declares personal and travel payments from Abbvie and Sanofi, outside submitted work. Y. W. Yew declared no COI. Z. Zhao is a speaker/advisor for and/or has received research funding from Abbvie, Astra Zeneca, Astellas, Novartis, Pfizer, Takeda, Sanofi, Lilly, Galderma, Janssen, GSK, BAYER, LEO, MEDA Pharma and ALK Pharma outside the submitted work. R. Zwiener declares no COI. M. Worm reports grants and personal fees from Stallergens, HAL Allergie, Bencard Allergie, Allergopharma, ALK‐Abello, Mylan Germany, Actelion Pharmaceuticals Deutschland, Biotest, AbbVie Deutschland, Lilly Deutschland Aimmune, DBV Technologies, Regeneron Pharmaceuticals, Sanofi Aventis, Leo Pharma, Novartis and Viatris, outside the submitted work.

## Data Availability

Data sharing not applicable to this article as no datasets were generated or analysed during the current study.
